# Are parents and adolescents in agreement on reporting of recurrent non-specific low back pain in adolescents? A cross-sectional descriptive study

**DOI:** 10.1186/s12887-015-0518-1

**Published:** 2015-12-08

**Authors:** Matthew Chiwaridzo, Nirmala Naidoo

**Affiliations:** Rehabilitation Department, College of Health Sciences, University of Zimbabwe, P.O Box A178, Avondale, Harare Zimbabwe; Division of Physiotherapy, Department of Health and Rehabilitation, Faculty of Health Sciences, University of Cape Town, Cape Town, South Africa

**Keywords:** Adolescents, Recurrent non-specific low back pain parents

## Abstract

**Background:**

Non-specific low back pain is a prevalent symptom in adolescents and is recurrent in some instances. Recent studies have highlighted the marked impact the condition has on daily life of adolescents. However, it is unclear if parents of adolescents reporting recurrent non-specific low back pain know about their child’s status. The purpose of the study was to determine the level of agreement between adolescents and their parents in reporting recurrent non-specific low back pain in Harare, Zimbabwe.

**Methods:**

This cross-sectional study formed part of a large study carried out to ascertain the prevalence of non-specific low back pain in Zimbabwean adolescents. Six hundred and twenty (*n* = 620) Medical Health Questionnaires were sent to parents. School-children with returned questionnaires and informed consents signed were subsequently eligible to participate. A reliable and validated low back pain study questionnaire was administered to 544 adolescents between the ages of 13 and 19 years randomly selected from government-administered schools. The questionnaire sought to determine adolescents with recurrent NSLBP. The Kappa statistic (k) was used to analyse agreement between adolescents and parental reports on recurrent NSLBP status.

**Results:**

Parental and school-children response rates were acceptable (90.3 and 97.8 %, respectively). The prevalence of recurrent NSLBP was 28.8 % [95 % Confidence Interval, CI = 26.0–31.6]. Both sexes were equally affected [*χ*^*2*^*(1) =*0.19*, p =* 0.67]. The prevalence increased with age in both sexes [*χ*^*2*^_trend_ =90.9, *p* < 0.001]. Parental reports agreed in 16.3 and 98.7 % for the adolescents with and without recurrent NSLBP respectively. The value of kappa (k) was 0.20 [SE = 0.04; 95 % CI, 0.13–0.27] with a prevalence index and bias index of −0.65 and 0.23, respectively. These results suggest poor strength of the agreement.

**Conclusions:**

Recurrent non-specific low back pain is relatively common among Zimbabwean adolescents. Most of the parents of school-children with recurrent non-specific low back pain are unaware of the low back pain status of their children. Although this does not dismiss the relevance of non-specific low back pain reported during adolescence, these findings create a need to involve parents in awareness or preventive initiatives against low back pain in schools.

**Electronic supplementary material:**

The online version of this article (doi:10.1186/s12887-015-0518-1) contains supplementary material, which is available to authorized users.

## Background

The adolescence period forms an important developmental stage in life [1]. The advances in medical care and health technology over the years have led to momentous changes in adolescent health worldwide. There has been a shift from injuries and communicable diseases to non-communicable and lifestyle-related conditions as prominent causes of disability adjusted-life years among adolescents. In the present society, adolescents are now vulnerable to conditions previously considered to be for adults only such as low back pain.

Non-specific low back pain (NSLBP) has become a common health problem in adolescents as in adults [2–4]. Lifetime prevalence rates approach those reported in adult studies [5]. Most cases of adolescent NSLBP are benign [6]. However, a subset of adolescents (13–36 %) experience severe and regular NSLBP commonly referred to as recurrent NSLBP [6–8]. These adolescents are likely to suffer significant health consequences [6]. Recurrent NSLBP have been found to be associated with seeking medical treatment, some degree of functional consequences, psychological distress, reduced health-related quality of life and school absenteeism in adolescents [6, 9–12]. Additionally, prospective studies link adult chronic low back pain to recurrent symptoms that began in adolescence [13–15]. This implies that adolescents with recurrent NSLBP constitute an important group of high risk adolescents warranting continued monitoring.

Given the potential impact recurrent NSLBP has in adolescents’ daily lives, activities and school, it should be a cause of concern not only to the school children, health-care professionals, and teachers but to parents or guardians as well. It is particularly crucial for parents or guardians to be aware of the low back pain status of their child, especially the recurrent type, for a number of reasons. Medical treatment for the condition can be sought early therefore preventing the debilitating effects of the condition. Monitoring and preventative efforts to minimise recurrent NSLBP can be sustained if all important stakeholders (parents, teachers, health care professionals, health-policy makers) are aware of the existence of the condition. Parents are often omitted in preventative initiatives against the condition and are often misinformed of the nature of the condition. Few studies have attempted to corroborate adolescents self-report of pain with parental reports [10, 16]. In Zimbabwe, to the authors’ knowledge, there is no data with regards to this matter. This is a significant shortcoming against a background of high prevalence rate of recurrent NSLBP in adolescents reported in a previous study [12]. Data on parents and adolescents level of agreement on reporting recurrent non-specific low back pain in adolescents would be useful in understanding the gravity of the condition in adolescents in light of the consequences reported in literature. Therefore, the main objective of this study was to examine the level of agreement between adolescents’ and parental reports on recurrent NSLBP.

## Methods

This study formed part of a large study conducted in two continuous phases, firstly, to determine the prevalence of adolescent recurrent NSLBP and secondly to ascertain the individual risk factors associated with the condition among adolescents in government administered secondary schools in Harare, Zimbabwe. Participants with recurrent NSLBP had to report pain which had occurred at least two times over the past year with each episode of lasting at least 24 h, with pain intensity of greater than two on the visual analogue scale (VAS) with at least a 30-day pain free period between the episodes. The methodology of the first phase has been described extensively elsewhere [17]. This article present on the findings on the level of agreement between adolescent and parental reports on recurrent NSLBP.

Briefly, a cross-sectional survey incorporating full-time secondary school students in Form One to Six between the ages of 10 and 19 years was conducted. The World Health Organisation (WHO) definition of an adolescent was adopted [18]. At the time of the study, there were 71 458 school-children in the 55 government administered schools in Harare. As the primary outcome was prevalence of recurrent NSLBP, minimum sample size of 495 was calculated using Epi Info Stalcalc based on the following parameters: regional prevalence of 13.5 % [19], a precision effect of 3 %, a design effect of 1 and 95 % confidence interval.

Schools and participants were recruited using a two-stage cluster sampling method. Secondary schools in Harare are classified by location into high-density suburbs schools (*n* = 17) and low-density suburbs schools (*n* = 38). Considering proportion between the clusters, one school was randomly selected from low density suburbs and two schools from the high density suburbs. In the second stage of sampling, one class was randomly selected at each level from Form One to Six from each participating school. All the students in the selected classes were then eligible to participate. A total of 620 school-children were eligible. However, school-children between 10 and 19 years and willing to participate in the study after being given parental approvals and were present on the day of the survey were included in the study. Students with parental reports of spinal pathologies or orthopaedic conditions, history of trauma to the back, central or peripheral nervous system problem and any overt or covert physical deformity including leg length discrepancy or scoliosis were excluded in the study.

Medical Research Council of Zimbabwe [ref: MRCZ/B/356] and the Human Research Ethics Committee [ref: HREC/189/2012] of the University of Cape Town gave ethical approval for the study. Institutional approval was obtained from Ministry of Primary and Secondary Education, Harare Provincial Education Offices and from school heads of the selected schools. School-children who volunteered to participate in the study were given information letters, adolescent medical health questionnaires and informed consent forms for parents to complete at home. Parental questionnaires were coded similarly with school-children low back pain study questionnaires for identification purposes. For confidentiality purposes, parental documents were sent sealed in an envelope and students were requested to return them in a provided sealed envelope. To minimise conversations between school-children and parents that will increase the percentage of agreement, the school children were not told at this stage that the study was about their low back pain status and whether their parents knew about it. Moreover, parents were specifically requested in the information letter to answer the adolescent medical health questionnaire truthfully and to the best of their knowledge without asking their child for input on the condition. The parental documents were to be returned to the school-form teachers within seven days. Within this time period, the researcher (MC) held meetings in person with parents to explain the rationale of the study and to address their concerns in the participating schools. The actual dates for the meetings and researcher personal contact details were specified in the parent information letters.

### Adolescent medical health questionnaire

The Adolescent Medical Health Questionnaire was adopted from Fanucchi et al. [20] study and modified to suit the local context (see Additional file 1). The 10 items on the questionnaire provided the medical history of school-children as reported by parents. Parents were defined as either biological or guardians living with the child at home. Adolescents with parental reports of spinal pathologies, deformities, fractures and neurological conditions were excluded [18]. A specific question to confirm for the adolescent recurrent NSLBP was asked as “In the past 12 months, has your child ever complained to you or any other family member at least twice of pain in the lower part of the back which lasted at least a day, not related to their menstrual cycles in females?” This question enabled direct comparisons to be made with adolescent report of recurrent NSLBP.

### Adolescent low back pain questionnaire

Adolescents who returned the medical health questionnaire fully completed and parental informed consent signed were considered eligible for the study. Every participant completed a detailed 22-item questionnaire (see Additional file 2) with questions pertaining to demographic data, recurrent NSLBP, characteristics of recurrent NSLBP (pain intensity, frequency, and duration), consequences (health-seeking behaviour, school absenteeism, functional limitations) and risk factors (smoking, school-bag use, sport participation, and sedentary lifestyle). Recurrent NSLBP was specifically defined as low back pain which had occurred at least two times over the past year with each episode of lasting at least 24 h, with pain intensity of greater than two on the VAS with at least a 30-day pain free period between the episodes [21]. It was asked regarding the last 12 months.

Data collection was conducted between June 2012 and December 2012 in the classrooms during school-hours in the presence of the school teacher and the researcher (MC). The students were instructed to sit approximately 50 cm apart to avoid deliberations between them. To facilitate understanding, the researcher read the questionnaires aloud to students in the lower classes (Form One and Two). The researcher attended to the participating schools consecutively during the second term of the academic year. Prior to use, content validity was determined by panel of five experts in field of epidemiology and musculoskeletal adolescent health. Experts had to evaluate each question/item on a four-point scale based on a criterion that considered four factors of relevance, clarity, simplicity and ambiguity [22]. Questions were then refined or discarded following the recommendations proposed by the content experts.

In a preliminary study, reliability of the English version of the questionnaire was evaluated among 40 final year students who completed the questionnaire on two separate occasions with a week interval. During the initial test, students were not informed about the re-test. The mean age of the respondents was 16.3 years (SD = 1.67) with 62.5 % of the respondents being females. Percentage agreement for the demographic details (age, gender, place of residence) was consistent between the test and re-test. For the primary outcome measure of recurrent NSLBP, the kappa coefficient (k) was moderate at 0.51.

### Statistical analysis

Statistica version 11 was used to analyse data gathered. Parametric tests were used to describe the data largely because of the large sample size even though some of the variables were not normally distributed [23]. However, Kolmogorov-Smirnov and Lilliefors tests were used to confirm normality of continuous data. Means and standard deviations (SD) were used to describe continuous data. Frequencies were used for categorical data. Recurrent NSLBP was expressed as a percentage of the total population. Exact 95 % confidence intervals (CI) were provided. Pearson’s chi-square test (*X*^2^) was used to evaluate the effect of gender on recurrent NSLBP prevalence at *p* ≤ 0.05. For analysis of agreement between the school-child and parent reports on recurrent NSLBP status, the kappa statistic (k) was used. The kappa statistic was interpreted based on a criteria provided by Landis and Koch [24]. A kappa statistic of 1 represents perfect agreement whereas 0 represents an agreement expected by chance [10]. Questionnaires with at least three variables missing were regarded as missing data and were discarded from the analysis.

## Results

**Fig. 1:**
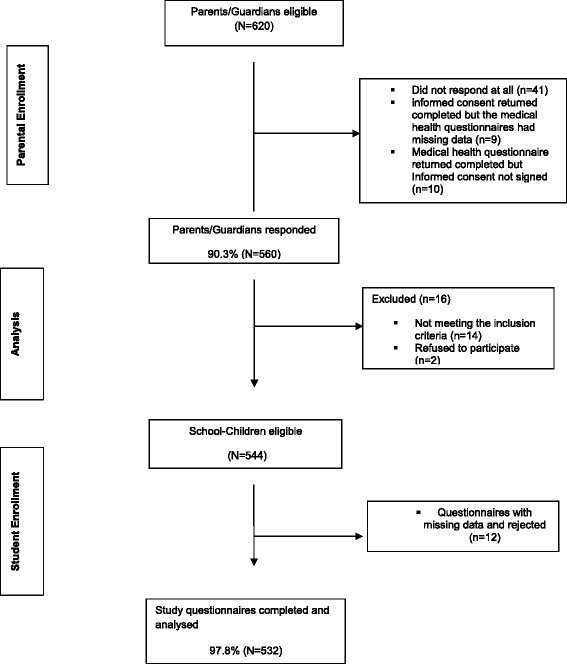
Flow chart depicting response rates of participants in the study

Figure 1 indicates that parental and school-children response rates were high (90.3 and 97.8 % respectively). The demographic characteristics of the study participants are presented in Table 1. The mean age of the sample was 16 years [SD = 1.72, range 13–19 years]. Female students constituted 53.8 % (*n* = 286) of the total sample. However, male students were significantly older compared to the female students [*t* (530) =2.34, *p* = 0.02].

**Table 1:** The demographic characteristics of the study participants (*n* = 532)

Characteristic	Total	%	Cumulative %
Females	286	53.8	
Males	246	46.2	
Age groups (years)			
13	29	5.4	5.4
14	98	18.4	23.8
15	101	19.0	42.8
16	96	18.0	60.8
17	90	16.9	77.7
18	67	12.6	90.2
19	51	9.6	100.0
Form (Years of education)			
1 (8)	74	13.9	13.9
2 (9)	116	21.8	35.7
3 (10)	106	19.9	55.6
4 (11)	100	18.8	74.4
5 (12)	77	14.4	88.8
6 (13)	59	11.2	100

**Fig. 2:**
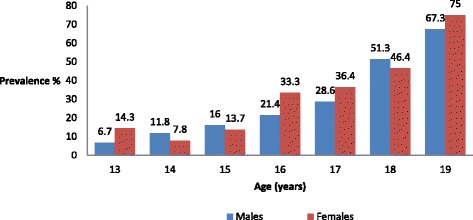
Prevalence of recurrent non-specific low back pain by age and gender

Based on the adolescent low back pain questionnaire, the prevalence of recurrent NSLBP for the past 12 months was 28.8 % (*n* = 153) [95 % Confidence Interval, 27.8–31.6]. Both sexes were equally affected [*χ*^*2*^*(1) =*0.19*, p =* 0.67]. However, Fig. 2 shows that prevalence of recurrent NSLBP in adolescents increased with increasing age in both sexes [*χ*^*2*^_trend_ =90.9, *p* < 0.001]. The majority of school-children with recurrent NSLBP (*n* = 82, 53.6 %) experienced more than three episodes in last 12 months. However, 85.6 % reported an episode to last less than seven days. Twenty-seven percent of the school-children with recurrent NSLBP sought medical treatment for the symptoms. About 21 % of the school-children with recurrent NSLBP reported sciatic symptoms.

### Agreement between adolescent and parental reports of recurrent NSLBP

Parental responses to the question “In the past 12 months, has your child ever complained to you or any other family member at least twice of pain in the lower part of the back which lasted at least a day, not related to their menstrual cycles in females?” were analysed for agreement against adolescents reports of recurrent NSLBP. Parental reports agreed in 16.3 and 98.7 % for the adolescents with and without recurrent NSLBP respectively (Table 2). The value of kappa was 0.20 [Standard Error, SE = 0.04; 95 % Confidence Interval, 0.125–0.272] with a prevalence index and bias index of −0.65 and 0.23 respectively. These results suggest that the strength of the agreement was poor. In spite of this slight agreement, parents were more likely to report that their child had recurrent NSLBP if the adolescent had reported sciatica [*χ*^*2*^*(1)* =4.33, *p* =0.04] but not medical treatment for the recurrent symptoms of low back pain [*χ*^*2*^*(1)* = 1.29, *p* = 0.26].

**Table 2:** Agreement between child and parental reports of recurrent non-specific low back pain (*n* = 532)

Characteristic	Responses	Parental report of adolescent recurrent NSLBP		
		Yes (%)	No (%)	Total
Adolescent recurrent NSLBP	Yes	25 (16.3)	128 (83.7)	153
	No	5 (1.3)	374 (98.7)	379
	Total	30	502	532

## Discussion

This study was designed to examine the level of agreement between parental and adolescents reports of recurrent NSLBP. The response rate from both parents and adolescents was satisfactory; a finding comparable with other cross-sectional studies in the literature [10, 18]. Bias due to non-participation could not have influenced the observed results. The self-administration of the study questionnaires to adolescents in structured environments (schools) could have had a positive impact. In addition, parents were informed of the study having had had formal approval from the Ministry of Education and school principals. This could have encouraged them to participate in a school-based project that evaluated the health of their school-child.

Literature specifically on recurrent NSLBP in adolescents is sparse. The few studies available have relied on different contextual definitions rendering comparisons between studies difficult. The present study uniquely relied on a definition of recurrent NSLBP agreed upon by experts in field of low back pain [17, 21]. The definition objectively stipulates the most important parameters that characterise recurrent NSLBP such as intensity, frequency, and duration of episodes. However, its validity has not been evaluated in adolescents [21].

Recurrent NSLBP is relatively common among Zimbabwean adolescents; a finding consistent with previous reports from other countries such as England and Netherland [6, 7]. The prevalence approached adult figures by end of adolescence [17]. Results of the first phase of the study have been described extensively elsewhere [17]. However, these findings are disturbing considering the strong link that exists between adolescent recurrent NSLBP and adulthood chronic non-specific low back pain [13]. From a public health perspective, these findings are worrisome and should stimulate concern in teachers, health professionals and parents. However, because of the cross-sectional design of the study and reliance on self-reports over a recall period of 12 months, the present findings may be interpreted with caution. No medical or radiological examinations were conducted to confirm self-reported recurrent NSLBP by adolescents. Nevertheless, pain has been described as a subjective phenomenon hence self-reports have been regarded as a valid method of assessing pain [16, 25].

The majority of recurrent NSLBP cases as reported by adolescents were not known by parents (83.7 %). Similarly, a cross-sectional study investigating the occurrence and characteristics of NSLBP among 1 446 adolescents in England observed a moderate agreement (k = 0.33) between school-children and their parents reports of the child’s condition [10]. Amongst the school-children reporting and not reporting NSLBP, parental reports agreed in 33 and 95 % of cases, respectively [10]. These findings raise fundamental questions regarding the significance of recurrent NSLBP self-reported by adolescents.

A number of reasons have been postulated to account for this unexpected anomaly. Interplay of parental and child-related factors partly explain the lack of agreement. Parents may forget about their child’s low back pain symptoms or interpret them as inconsequential [10, 16]. In addition, the condition may not be severe enough for adolescents to inform their parents [10]. The majority of the adolescents with recurrent NSLBP failed to seek medical treatment for the condition. Fear of admonishment by parents may also have caused adolescents to withhold information from parents. In Zimbabwe, anecdotal beliefs link adolescent low back pain complaints to socially unacceptable behaviours such as early sexual indulgence [12]. This possibly contributes to the lack to the agreement between the reports. Additionally, knowledge of the potential to incur costs to parents for medical care could be another possible explanation considering the socio-economic challenges in the country.

Interestingly, the present study indicated that parents knew about their child’s recurrent NSLBP status if the child had reported sciatica but not medical treatment. Although sciatica is regarded as an important indicator of severe and continuous low back pain, these findings should be interpreted with caution [26]. In the Zimbabwean context, parents are responsible for arranging consultation and payment for the medical services in case their child needs a health professional. Parents should have been aware of their child’s recurrent NSLBP status for the adolescents who sought medical treatment. Therefore, these findings possibly indicate that school-children sought medical care without parental knowledge. The fact that sciatica was associated with parental knowledge of child recurrent NSLBP status suggests a possible existence of various other musculoskeletal problems such as lower limb muscular or joint pain which possibly warranted medical treatment.

### Limitations

This study had limitations which included reliance on self-reported data from parents and adolescents. It is possible for the participants to forget the exact nature and characteristics of the recurrent NSLBP considering the information was collected retrospectively. The accuracy of the responses from both participants could have been affected by recall bias thereby over or under-estimating the level of agreement especially considering the recall period of 12 months used in the study. On the other hand, it is possible that the parents and school-children could have discussed the study extensively between the time the school-children brought the parental documents home to the time they completed their own low back pain questionnaire at school few days later. This could have affected the level of percentage of agreement. In addition, the study sample for adolescents was not representative of all the adolescents in schools in Harare, Zimbabwe. Only three secondary schools were randomly selected from a list of government administered schools.

## Conclusion

Recurrent NSLBP is relatively common among school-aged Zimbabwean adolescents. The reported recurrent NSLBP at the end of the adolescent period approaches that reported for adults. Both male and female students are equally affected. Most of the parents are unaware of the recurrent NSLBP status of their children. This raises concerns regarding the significance of the condition in adolescence but also create a need for raising awareness of the condition among parents. Parents knew about their child’s recurrent NSLBP status if the child had reported sciatica. Future studies are needed to determine the existence of various other musculoskeletal problems such as lower limbs muscular or joint pains among adolescents with low back pain.

## Additional files

Additional file 1:
**Adolescent medical health questionnaire.** (DOCX 34 kb) Additional file 2:
**Adolescent low back pain questionnaire.** (DOCX 162 kb)
