# Supplementary material for: Are parents and adolescents in agreement on reporting of recurrent non-specific low back pain in adolescents? A cross-sectional descriptive study
Source: BMC Pediatr. 2015 Dec 8;15:203. doi: 10.1186/s12887-015-0518-1 (PMC4673738; doi:10.1186/s12887-015-0518-1)
Supplement: Additional file 1: — Adolescent medical health questionnaire. (DOCX 34 kb) [file 12887_2015_518_MOESM1_ESM.docx]

**Adolescent medical health questionnaire Code number………...**

**Instruction:** Please indicate with a tick (√) in the appropriate box.

1. In the past 12 months, has your child ever complained to you or any other family member at least twice of pain or discomfort in the lower part of his/her back, which lasted a day or longer, not related to their menstrual cycle in females?

Yes No

1. Is there anyone in the family (including yourself) that you know of who complains of pain in their lower part of the back and takes medication or used to take medication for that?

Yes No

If Yes, what is the relationship to the child……………………………………............

1. Has your child sustained any injury to his/her back either at home or at school that you are aware of?

Yes No

1. Has your child been diagnosed by a doctor or physiotherapist with any condition that affect his/her back?

Yes No

If Yes, Please list the condition………………………………………………………..

1. Is your child currently following a specialised treatment programme with a medical doctor for pain he/she feels in the lower part of his/her back?

Yes No

1. Is your child currently following a rehabilitation programme after an operation on his/her bones, ligaments, muscles and tendons of his/her limbs and trunk?

Yes No

If Yes, please would you list………………………………………………………..

1. Does your child find it difficult or impossible to stand up on his/her own without using an assistive device for example a crutch?

Yes No

1. Has your child been diagnosed with a neurological condition which affects the tone in his/her muscles?

Yes No

If yes, please list: …………………………………………………………………

1. Does your child have a visible problem with his/her back (scoliosis) that requires him/her to wear a brace or to go for an operation?

Yes No

1. Has your child been diagnosed with a spinal tumour or unequal legs at any time since birth?

Yes No
