# Supplementary material for: Are parents and adolescents in agreement on reporting of recurrent non-specific low back pain in adolescents? A cross-sectional descriptive study
Source: BMC Pediatr. 2015 Dec 8;15:203. doi: 10.1186/s12887-015-0518-1 (PMC4673738; doi:10.1186/s12887-015-0518-1)
Supplement: Additional file 2: — Adolescent low back pain questionnaire. (DOCX 162 kb) [file 12887_2015_518_MOESM2_ESM.docx]

**Low back pain questionnaire Code number……………**

**Date of Birth** ……../………../………..

**Gender**  Male Female

**What Form are you in? .........................................**

**Where do you stay? …………………………….**

**Section A: Information on Low Back Pain**

1. Have you ever experienced pain or discomfort in the lower part of your back which lasted for one day (24hrs) or longer in your life (**not associated with menstruation in females**)

Yes No

1. How old were you when you first experienced that pain in the lower part of your back? ..............................years old.

**The next questions will all be based on your experience of low back pain in the past 12 months. Please answer based ONLY on the last 12 months.**

1. For the last 12 months, did you **EVER** have pain in the lower part of your back?

Never Once Frequently

**NB: If you answered, frequently, Please continue with questions below. If you did not indicate frequently, please go to question number 8.**

1. How **OFTEN** would you say that you have experienced pain episodes in your lower part of your back, on average, during the last 12 months?

Twice Thrice More than Thrice

1. How long (in days) does your lower back pain episode **usually** last, in the past 12 months?

1-7 8-14 15-21 22-29 ≥1month

1. How severe or serious, **on average**, is the pain that you frequently feel in the lower part of your back on a scale of 0 to 10? 0 means no pain and 10 means worst pain ever felt in your life. (**Please mark with an X on the figure below, adopted from Ibrahim [27].**

1. Do you experience any pain in your buttock; thigh, calf and foot that appear to be associated with the pain you frequently feel in your lower back?

Yes No

1. Do you have the pain in the lower part of your back **NOW?**

Yes No

**NB: If you answered, YES to the question above, Please answer the following question. If you did not, please, skip to number 10.**

1. How severe or serious is the pain you feel **NOW** in the lower part of your back, on average, using a score of 0 to 10? 0 indicates no pain or discomfort and 10 indicate the worst pain or discomfort you ever felt in your life (**Please mark with an X on the figure below, adopted from Ibrahim [27]**

1. Have you ever sought treatment for low back pain or information from any medical personnel or traditional healers regarding the pain that you frequently feel?

Yes No

1. Has your pain in the lower back ever interfered with any of the following activities? Please indicate with (√) your response.

| Sitting on a school-chair for a 30 minutes | Yes/ No |
| --- | --- |
| Reaching up to get a book from a high shelf | Yes/No |
| Standing over a long time at school/home | Yes/No |
| Walking over a long distance | Yes/ No |
| Participating in sports/exercise at school/home | Yes/No |
| Bending down to put socks on | Yes/No |
| Carrying your school bag to school | Yes/No |
| Sitting up in bed from lying position | Yes/No |
| Running fast for class or catching a bus/car | Yes/No |

1. Have you **EVER** missed school for at least one day (school absenteeism) because of your low back pain?

Yes No

**Section B**: **Schoolbag information. Please tick (√) where appropriate.**

1. Do you carry a school bag to school?

Yes No

1. How would you **PERCEIVE** the weight of your school bag that you carry to school?

Heavy Average weight Not Heavy

1. On average, how long (minutes) do you normally spend carrying your schoolbag **TO** and **FROM** school?

< 5min
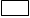
 5 – 10min
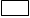
 11 – 20min
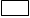
 21 – 30min
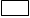
 >30min
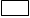


1. How do you **normally** carry your school-bag to and from school?

Right/ Left Shoulder Over both Shoulders Right/Left Hand

**Section C: Sports participation.**

1. Do you play any sport or exercise regularly? (Either at school or at home)

Yes No

1. If **YES**, Please list the sport(s) or exercise(s) activities that you play at school or home **a)…………………**……………………….**.b)…………………………………………**
2. How many **HOURS** a week in total, do you play sport/exercise at school or at home?

<2 hrs.
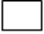
 2-<4 hrs.
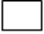
 4-<6 hrs.
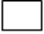
 6-<10 hrs.
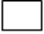
 ≥10 hrs.
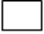


1. About how long, on average, do you spend sitting per day, including weekends, watching television, playing video games or using laptop/computer, listening to music?

<2 hrs.
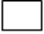
 2-<4 hrs.
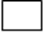
 4-<6 hrs.
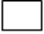
 6-<10 hrs.
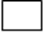
 ≥10 hrs.
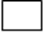


**Section D: Smoking status**

1. Have you **EVER** smoked cigarettes in your life?

Yes No

1. Did you at least smoke **ONE** cigarette in the past week?

Yes No
